# Xin-Fu-Kang oral liquid improves cardiac function and attenuates miR-223–associated NF-κB/NLRP3 pyroptotic signaling in chronic heart failure

**DOI:** 10.3389/fphar.2025.1697422

**Published:** 2025-12-11

**Authors:** Zelin Ye, Mingrui Liu, Xiaohan Zhang, Huaqin Wu, Zhiling Qiu, Ruoning Chai, Yuanhui Hu

**Affiliations:** 1 Department of Cardiology, Guang’anmen Hospital, China Academy of Chinese Medical Sciences, Beijing, China; 2 Data Center of Traditional Chinese Medicine, China Academy of Chinese Medical Sciences, Beijing, China; 3 Department of General Internal Medicine, Guang’anmen Hospital, China Academy of Chinese Medical Sciences, Beijing, China

**Keywords:** Xin-Fu-Kang, chronic heart failure, pyroptosis, miR-223, NF-κB, NLRP3

## Abstract

**Background:**

Pyroptotic signaling involving nuclear factor-kappa B (NF-κB) and NOD-like receptor family pyrin domain–containing 3 (NLRP3) has been implicated in chronic heart failure (CHF). Xin-Fu-Kang (XFK) is a nine-herb formula used clinically for CHF with “qi deficiency and blood stasis.” Although cardioprotective effects have been reported, it remains unclear whether XFK modulates myocardial pyroptotic signaling via miR-223–dependent regulation of NF-κB.

**Methods:**

A CHF model was established by permanent left anterior descending coronary artery (LADCA) ligation in rats, and an *in vitro* oxygen–glucose deprivation/reoxygenation (OGD/R) injury model was generated in H9c2 cardiomyocytes. Cardiac structure and function were assessed by transthoracic echocardiography and histology. Myocardial inflammation and pyroptotic signaling were quantified by ELISA for interleukin-1 beta (IL-1β) and interleukin-18 (IL-18), and by immunoblotting for NLRP3, pro-caspase-1/caspase-1 ratio, Apoptosis-associated speck-like protein containing a CARD (ASC), cleaved gasdermin D N-terminal fragment (GSDMD-N), and NF-κB p65 phosphorylation. Nuclear–cytoplasmic fractionation and immunofluorescence tracked p65 translocation. Causality was probed by miR-223 gain- and loss-of-function, with functional rescue using a miR-223 inhibitor. RT-qPCR was used to measure the mRNA levels of *NF-κB p65* and miR-223.

**Results:**

LADCA produced marked systolic dysfunction with chamber dilation, increased myocardial IL-1β and IL-18, increased NLRP3, ASC, GSDMD-N, and p65 phosphorylation, and decreased the pro-caspase-1/caspase-1 ratio. XFK improved cardiac function and structural integrity, attenuated fibrosis and cardiomyocyte apoptosis, reduced inflammatory cytokines, and diminished NLRP3 and ASC abundance. In OGD/R-injured H9c2 cells, XFK preserved viability, limited lactate dehydrogenase release, decreased NLRP3, ASC, GSDMD-N, and IL-1β, increased the pro-caspase-1/caspase-1 ratio, and restrained NF-κB activation by reducing p65 phosphorylation and nuclear translocation. Mechanistically, XFK upregulated miR-223, and miR-223 overexpression reproduced the suppression of pyroptosis-related readouts linked to NF-κB/NLRP3 signaling. Inhibition of miR-223 attenuated the protective effects of XFK, supporting the interpretation that XFK-mediated modulation of NF-κB-related inflammatory signaling is at least partly dependent on miR-223.

**Conclusion:**

NF-κB–linked NLRP3 pyroptotic signaling represents a prominent feature in the CHF model examined. These findings suggest that XFK exerts protective effects in CHF via miR-223–dependent modulation of NF-κB/NLRP3 pyroptotic signaling, supporting its potential adjunctive strategy to mitigate inflammation-driven cardiac dysfunction.

## 1 Introduction

Chronic heart failure (CHF) remains a major global health burden, characterized by progressive cardiac dysfunction resulting from detrimental structural and functional changes (Roger, 2021). Pathological processes including persistent inflammation and cardiomyocyte death are critical drivers of cardiac remodeling and CHF progression (Aimo et al., 2020). Among cell death pathways, pyroptosis—a highly inflammatory form of programmed cell death mediated by inflammasomes, caspases, and the pore-forming protein gasdermin D (GSDMD)—has emerged as a significant contributor to myocardial injury and dysfunction in CHF by amplifying inflammation through the release of cytokines like IL-1β and IL-18 (Chai et al., 2022; Zhang Z. et al., 2024).

The nuclear factor-kappa B (NF-κB) signaling pathway is a central regulator of cardiac inflammation and plays a key role in promoting the activation of inflammasomes, such as NOD-like receptor family pyrin domain–containing 3 (NLRP3), thereby linking inflammation to pyroptosis (Zhang Z. et al., 2024; Xu et al., 2025; Xiong et al., 2024). MicroRNAs (miRNAs) act as crucial regulators of gene expression, and microRNA-223 (miR-223) has been specifically implicated in modulating inflammatory responses, partly through its ability to target components of the NF-κB pathway and NLRP3 inflammasome signaling (Haneklaus et al., 2013; Yan et al., 2019; Pan et al., 2022). Given the involvement of miR-223 in cardiovascular inflammation and NF-κB’s role in pyroptosis, the miR-223/NF-κB axis has been proposed as a potential therapeutic target for modulating myocardial pyroptosis in CHF.

Traditional Chinese Medicine (TCM) defines “blood stasis” as a pattern characterized by impaired blood circulation and pathological accumulation, often involving microvascular dysfunction and hypercoagulability, which worsen tissue injury in cardiovascular diseases (Yu and Wang, 2014; Junxiu et al., 2017; Xin et al., 2021). The phenomena resulting from pyroptosis-driven inflammation in CHF—specifically, amplified myocardial injury (Shi et al., 2021), ventricular dysfunction (Piams et al., 2023; Qin et al., 2024), and the associated microcirculatory disturbances (Sun et al., 2021; Feng et al., 2021)—share significant conceptual parallels with the manifestations encompassed by the term “blood stasis.” The TCM understanding of “blood stasis” can serve as an explanatory lens to appreciate the detrimental impact of pyroptosis-related inflammatory events on the progression of CHF. Building upon this TCM understanding of pathological patterns like blood stasis, therapeutic strategies in TCM have traditionally aimed to address these hemodynamic and microcirculatory imbalances.

Xin-Fu-Kang (XFK) oral liquid is a nine-herb prescription created by senior TCM physicians to treat CHF presenting with “blood stasis and qi deficiency” (Zhang et al., 2025). Preclinical studies in CHF models demonstrate that XFK enhances ventricular performance and mitigates pathological remodeling, supporting cardioprotective potential. Previous mechanistic work showed that XFK regulates mitophagy and energy metabolism in models of post-ischemic injury via the PI3K/AKT pathway (Zhang et al., 2025; Qiu et al., 2018), and that its components can ameliorate inflammation, oxidative stress, and calcium dyshomeostasis in CHF. While these findings highlight XFK’s benefits, which are potentially linked to mitochondrial function (Zhang et al., 2025), its specific impact on myocardial pyroptosis—another critical inflammatory process in CHF—remains unexplored. Furthermore, whether the miR-223/NF-κB axis is involved in the effects of XFK on pyroptosis has not been investigated.

Therefore, we hypothesized that XFK may improve CHF by mitigating myocardial pyroptosis through miR-223–associated modulation of NF-κB/NLRP3-related inflammatory signaling. The present study aimed to evaluate the effects of XFK on cardiac function and myocardial pyroptosis in a rat model of CHF and to examine whether miR-223–linked regulation of the NF-κB/NLRP3 pathway contributes to these effects.

## 2 Methods

### 2.1 Preparation of XFK

XFK is a polyherbal preparation composed of *Astragalus mongholicus* Bunge [Fabaceae; Astragali radix; 30 g; Huangqi], *Salvia miltiorrhiza* Bunge [Lamiaceae; Salviae miltiorrhizae radix et rhizoma; 30 g; Danshen], *Angelica sinensis* (Oliv.) Diels [Apiaceae; Angelicae sinensis radix; 25 g; Danggui], *Ligusticum chuanxiong* Hort. [Apiaceae; Chuanxiong rhizoma; 25 g; Chuanxiong], *Citrus × aurantium* L. [Rutaceae; Aurantii fructus immaturus; 25 g; Zhishi], *Ganoderma sichuanense* J.D.Zhao & X.Q.Zhang [Ganodermataceae; Ganoderma; 25 g; Lingzhi], *Panax ginseng* C.A.Mey. [Araliaceae; Ginseng radix et rhizoma; 10 g; Renshen], *Epimedium brevicornu* Maxim. [Berberidaceae; Epimedii folium; 20 g; Yinyanghuo], and *Aconitum carmichaelii* Debeaux [Ranunculaceae; Aconiti lateralis radix praeparata; 10 g; Fuzi]. Scientific names with authorities and families were validated against *Plants of the World Online* and the *Medicinal Plant Names Services*, and pharmacopeial drug names follow the *Chinese Pharmacopoeia*.

All raw herbal materials used in XFK were sourced from the Pharmacy Department of Guang’anmen Hospital as a single manufacturing batch and authenticated by two pharmacognosists following the *Chinese Pharmacopoeia*, with voucher specimens retained. XFK was prepared as follows: the nine herbal components were decocted twice in water for 60 min each; the combined filtrates were cooled to 40 °C; ethanol (95%) was added slowly to a final concentration of 70% (v/v); the mixture was left to stand for 24 h; the supernatant was collected, and the liquid was concentrated under reduced pressure to yield a final extract at 3.2 g/mL crude-herb equivalents, in line with our previous protocol (Zhang et al., 2025). To convert crude-herb equivalents to a dry-extract basis, triplicate aliquots of the same batch stock were dried under reduced pressure at 60 °C to constant weight to determine total solids, giving an extraction yield of 25.0% (w/w; *n* = 3), corresponding to a drug–extract ratio (DER) of approximately 4:1. Accordingly, the dry-extract concentration of the stock was 0.8 g/mL (3.2 × 0.25). The stock was stored at 4 °C protected from light and freshly diluted with purified water to the target concentration on dosing days.

The phytochemical profile of XFK has been established previously using a UHPLC–Q-Exactive HF-X platform, including dual-polarity fingerprints (ESI+ and ESI–), data-dependent MS/MS acquisition, base-peak chromatograms, and the annotation of serum-absorbed prototype constituents (*n* = 120) with their chemical-class distribution (Zhang et al., 2025). The current study used the same pharmacy source and manufacturing protocol.

### 2.2 Drugs and reagents

The herbal medicines were purchased from Kangmei Pharmaceutical Co., Ltd: Huangqi (230200951); Danshen (221103151); Danggui (230304261); Chuanxiong (230701711); Zhishi (232004761); Lingzhi (230400631); Renshen (231102); Yinyanghuo (221104161); Fuzi (23010222). Other drugs and reagents included: Captopril (#H32023731, China); fetal bovine serum (#10099141, Gibco, United States); streptomycin (#T1320, Solarbio, China); penicillin (#P1400, Solarbio, China); Dulbecco’s Modified Eagle Medium (#D6570, Solarbio, China); Trypsin-EDTA (0.25%) (#T1300, Solarbio, China); Phosphate-Buffered Saline (PBS, #P1010, Solarbio, China); 4% Paraformaldehyde (#P1110, Solarbio, China); TdT-mediated dUTP Nick-End Labeling (TUNEL) Assay Kit (#C1086, Beyotime, China); CCK-8 Kit (#C0038, Beyotime, China); Lactate Dehydrogenase Cytotoxicity Assay Kit (#C0017, Beyotime, China); Annexin V-FITC/PI Apoptosis Detection Kit (#C1062M, Beyotime, China); RIPA Lysis Buffer (#R0010, Solarbio, China); PMSF (#P0100, Solarbio, China); Phosphatase Inhibitor Cocktail (#P1260, Solarbio, China); BCA Protein Assay Kit (#PC0020, Solarbio, China); TRIzol Reagent (#9109, Takara, Japan); PrimeScript™ RT Master Mix for mRNA (#RR036A, Takara, Japan); miRNA First-Strand cDNA Synthesis Kit (#MR101-01, Vazyme, China); SYBR Green qPCR Master Mix (#Q711-02, Vazyme, China); Lipofectamine 3000 Transfection Reagent (#L3000015, Thermo Fisher Scientific, United States); Nuclear and Cytoplasmic Protein Extraction Kit (#P0027, Beyotime, China); Bovine Serum Albumin (BSA, #A8020, Solarbio, China); Triton X-100 (#T8200, Solarbio, China); DAPI Staining Solution (#C1002, Beyotime, China); Rat IL-1β ELISA Kit (#RK00006, ABclonal, China); Rat IL-18 ELISA Kit (#PI555, Beyotime, China); ECL Chemiluminescence Detection Kit (#PE0010, Solarbio, China).

### 2.3 Animals and ethics statement

Male Sprague–Dawley rats (6 weeks old; 180–200 g) were purchased from Beijing Hua Fu Kang Bioscience Co., Ltd. After acclimation, animals were maintained in a temperature-controlled room (22 °C ± 2 °C) on a 12:12-h light–dark cycle, with unrestricted access to standard chow and water throughout the study. All procedures and husbandry were conducted in accordance with the Ethical Regulations for the Care and Use of Laboratory Animals of Guang’anmen Hospital and were approved by its Institutional Animal Care and Use Committee (IACUC, approval No. IACUC-GAMH-2021-020).

### 2.4 Animal model and interventions

One hundred and twenty male Sprague–Dawley rats were used. Acute myocardial infarction was produced by permanent ligation of the left anterior descending coronary artery (LADCA) as described previously (Pfeffer et al., 1979). Under sodium pentobarbital anesthesia, animals were endotracheally intubated and mechanically ventilated. A left thoracotomy was performed through the fourth intercostal space, the heart was exteriorized, and the LADCA was tied with 5–0 silk approximately 2 mm distal to the tip of the left atrial appendage. Sham animals underwent the same procedure except that the suture was placed but not tightened. Body temperature was maintained on a heating pad until recovery.

Of the 120 rats that underwent surgery, 94 survived the immediate perioperative period, defined *a priori* as the first 24 h after LADCA ligation or sham manipulation, yielding a postoperative survival of 78.3%. At 24 h after the operation, 90 survivors were randomly allocated to experimental cohorts, consisting of the sham group and the LADCA ligation subgroups, which comprised the model group, the captopril group, the low-dose XFK group, the medium-dose XFK group and the high-dose XFK group. Four additional survivors were not allocated and were not included in the analyses. There were no deaths during the subsequent 8-week dosing and follow-up period. Serum biochemical analyses showed no statistically significant differences in ALT, AST, or CREA levels among the sham group, the model group, and the dosing group (Supplementary Figure S1).

Dose selection was anchored to the clinical adult regimen and determined by body-surface-area (BSA) normalization. The clinical adult dose of XFK is 10 mL (1.6 g raw herbs/mL) three times daily (total 48 g/day for a 60 kg human). Using BSA conversion (Km_human = 37, Km_rat = 6; factor ≈6.2) (Pfeffer et al., 1985), the rat equivalent daily dose is 4.93 g/kg. For operational consistency with legacy batches and to achieve clean gavage volumes and dose spacing, we used 4.8 g/kg/day (−2.6%) as the reference (“1×”). Based on the 3.2 g/mL stock, the gavage volumes were approximately 1.5 mL/kg for the 1× level and 3.0 mL/kg for the 2× level, within standard feasibility for rats. With the batch-specific extraction yield of 25%, 4.8 g/kg/day (raw herbs equivalent) corresponds to 1.20 g/kg/day on a dry-extract basis; the low/medium/high levels thus equated to 0.60, 1.20, and 2.40 g/kg/day of dry extract, respectively. XFK or vehicle (purified water) was administered by oral gavage once daily for 8 weeks. Captopril was given at 10 mg/kg/day (p.o.), and vehicle was purified water.

### 2.5 Myocardial histology examination

Hearts were arrested in diastole with ice-cold KCl, rinsed, and fixed in 4% paraformaldehyde. Paraffin sections (6 µm) were prepared. Hematoxylin–eosin (HE) was used for general morphology; Masson’s trichrome for collagen; TUNEL for DNA fragmentation. Histological procedures were performed according to standard protocols as previously described (Chai et al., 2023). All quantitative analyses of the stained images were performed using ImageJ software.

### 2.6 Echocardiography

After 8 weeks of treatment, transthoracic echocardiography (Vevo 3100, VisualSonics) was performed under 1.5%–2.0% isoflurane. M-mode images at the papillary muscle level were used to measure left ventricular internal dimension at end-diastole (LVIDd) and left ventricular internal dimension at end-systole (LVIDs); ejection fraction (EF), fractional shortening (FS), and cardiac output (CO) were derived from three consecutive cycles and averaged.

### 2.7 Cell culture

H9c2 rat cardiomyoblasts (Cell Resource Center, PUMC, Beijing) were cultured in DMEM +10% FBS +1% penicillin/streptomycin at 37 °C in 5% CO_2_. XFK-medicated serum was prepared as follows. Forty male Sprague–Dawley rats (8 weeks, 280–300 g) were randomized to an XFK group and a vehicle group (*n* = 20 per group). The XFK group received XFK by oral gavage at the rat-equivalent dose, twice daily for 7 days; controls received water, volume-matched. Ninety minutes after the final dose, blood was collected from the abdominal aorta, allowed to clot at RT for 2 h, and centrifuged at 3,000 rpm for 10 min. Serum was heat-inactivated at 56 °C for 30 min, 0.22-µm filtered, aliquoted, and stored at −80 °C. Sera from each group were pooled and, immediately before use, diluted with DMEM/F12 to the indicated volume fraction for cell experiments. *In vitro* oxygen–glucose deprivation/reoxygenation (OGD/R) was used as the injury model: glucose-free DMEM under 95% N_2_/5% CO_2_ for 6 h, followed by reperfusion with high-glucose DMEM under normoxia for 24 h. For pharmacologic intervention, XFK was added 24 h before OGD and maintained during reperfusion. Controls remained in normoxic, glucose-containing medium.

### 2.8 Cell viability and apoptosis assays

Cell viability was assessed using a CCK-8 Kit according to the manufacturer’s instructions. H9c2 cells were seeded in 96-well plates at a density of 5 × 10^3^ cells/well. Following the designated treatments as described in the experimental model, 10 µL of CCK-8 solution was added to each well, and the plates were incubated for an additional 2 h at 37 °C. Cytotoxicity was evaluated by measuring the activity of lactate dehydrogenase (LDH) released into the culture medium from damaged cells, using an LDH Cytotoxicity Assay Kit. The rate of apoptosis was quantified by flow cytometry using an Annexin V-FITC/PI Apoptosis Detection Kit. After treatment, both adherent and floating cells were harvested and collected by centrifugation. The cells were washed twice with cold PBS and then resuspended in 1× binding buffer. Subsequently, cells were stained with 5 µL of Annexin V-FITC and 10 µL of Propidium Iodide (PI) for 15 min at RT in the dark.

### 2.9 Western blot

Total protein from ventricular tissue and H9c2 cells was extracted in RIPA buffer with PMSF and phosphatase inhibitors. Nuclear/cytoplasmic fractions were prepared using a commercial kit. Protein concentration was determined by BCA assay. Equal protein (30–50 µg/lane) was separated by SDS-PAGE and transferred to PVDF membranes. Membranes were blocked (5% nonfat milk or 5% BSA in TBST, 1.5 h, RT) and incubated with primary antibodies overnight (4 °C): NLRP3 (#ab214185, Abcam, United Kingdom); IL-1β (#16806-1-AP, Proteintech, United States); GSDMD (#ab209845, Abcam, United Kingdom); pro-caspase-1 (#ab179515, Abcam, United Kingdom); p65 (#10745-1-AP, Proteintech, United States); P-p65 (Ser536) (#3033, Cell Signaling Technology, United States); GAPDH (#60004-1-Ig, Proteintech, United States). HRP-conjugated secondary antibodies were applied for 1 h at RT; signals were developed by ECL and quantified by densitometry. For subcellular p65, β-actin and histone H3 were used as cytoplasmic and nuclear loading controls, respectively.

### 2.10 ELISA

Ventricular tissue was weighed and homogenized in ice-cold PBS. After centrifugation, supernatants were assayed for IL-1β and IL-18 using commercial ELISA kits, and absorbance was read at 450 nm.

### 2.11 RT-qPCR

Total RNA was isolated (TRIzol). To quantify NF-κB-related transcription level, *RELA* (p65) mRNA was measured using PrimeScript™ RT Master Mix and SYBR Green-based qPCR, whereas miR-223 was measured using a dedicated miRNA first-strand cDNA kit. qPCR was performed on a CFX96 real-time PCR system (Bio-Rad). Relative expression was calculated as 2^–ΔΔCt, with GAPDH used for mRNA normalization and U6 used for miRNA normalization (Supplementary Table S1).

### 2.12 Cell transfection

To investigate the functional role of miR-223, H9c2 cells were transfected with a miR-223 mimic (for overexpression), a miR-223 inhibitor, or their respective negative controls (NC). All small RNA oligonucleotides were synthesized by GenePharma (Shanghai, China). The transfections were performed using Lipofectamine 3000 Transfection Reagent according to the manufacturer’s protocol. Cells were seeded to reach 60%–70% confluency on the day of transfection. The small RNAs were transfected at a final concentration of 50 nM. Twenty-four hours after transfection, the cells were subjected to subsequent experiments, such as OGD/R injury and XFK treatment.

### 2.13 Immunofluorescence staining

To visualize the subcellular localization of NF-κB p65, H9c2 cells grown on glass coverslips were processed for immunofluorescence staining. Following the designated treatments, cells were fixed with 4% paraformaldehyde for 15 min and then permeabilized with 0.2% Triton X-100 for 10 min. After blocking with 5% BSA for 1 h at RT, the cells were incubated overnight at 4 °C with the primary antibody against p65. The next day, after washing with PBS, the cells were incubated with a FITC-conjugated goat anti-rabbit secondary antibody for 1 h in the dark. Finally, nuclei were counterstained with DAPI for 5 min. The coverslips were mounted onto slides, and images were captured using a laser scanning confocal microscope.

### 2.14 Statistical analysis

All quantitative data were expressed as the mean ± standard deviation (SD). Comparisons between two groups were analyzed using a two-tailed Student’s t-test. For comparisons among three or more groups, one-way analysis of variance (ANOVA) was performed, followed by Tukey’s multiple comparisons *post hoc* test. A P-value of less than 0.05 was considered statistically significant. All experiments were conducted with at least three independent biological replicates.

## 3 Results

### 3.1 Effect of XFK on cardiac function

**FIGURE 1:**
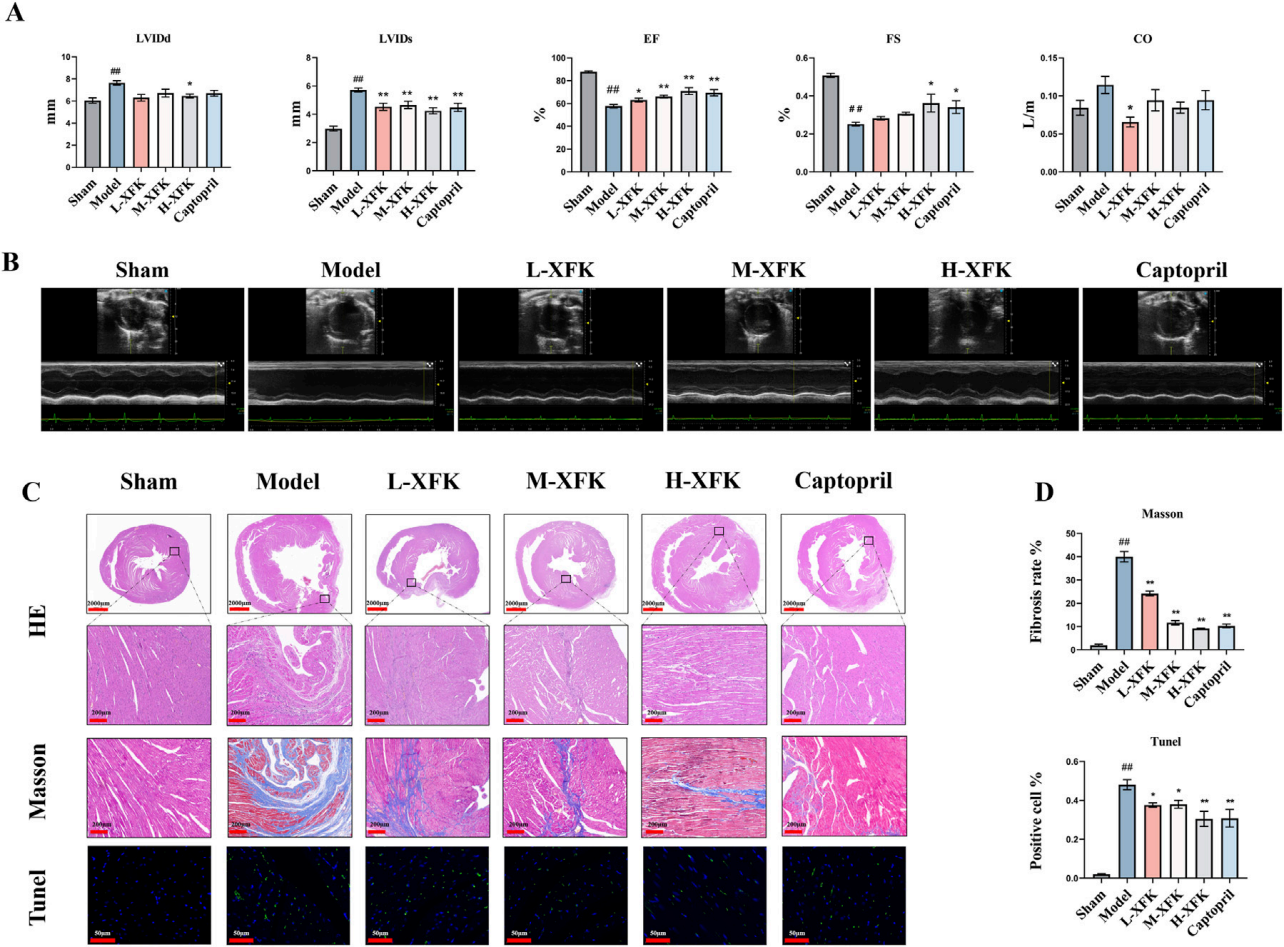
XFK alleviates ventricular dysfunction, fibrosis, and cardiomyocyte death in rats with CHF. **(A)** Representative M-mode echocardiograms (upper traces) and pooled quantitative indices of LVIDd and LVIDs, EF, FS, and CO, which were obtained 8 weeks after surgery. **(B)** Representative echocardiographic images. **(C)** HE staining shows preserved myofibre architecture in sham hearts, extensive fibre rupture and inflammatory infiltration in the model group (scale bar = 200 µm). Masson’s trichrome staining reveals marked interstitial and replacement fibrosis (blue) in the model myocardium that is progressively curtailed by XFK treatment (scale bar = 200 µm). TUNEL identifies sparse DNA-fragmentation-positive nuclei in sham hearts, abundant positivity in the model group, and a graded reduction with increasing XFK doses (scale bar = 50 µm). **(D)** Morphometric analysis quantify collagen area fraction (Masson) and the proportion of TUNEL-positive nuclei, expressed as percentages of total tissue area or nuclei. Data are mean ± SD. ##*P* < 0.01 vs. Sham; *P < 0.05, **P < 0.01 vs. Model. L/M/H-XFK (low-, medium-, and high-dose XFK).

Echocardiography (Figures 1A,B) demonstrated severe ventricular dilatation and systolic failure 8 weeks after LADCA ligation. LVIDd and LVIDs were markedly enlarged, whereas EF, FS and CO were markedly reduced (*P* < 0.05). Daily oral XFK produced a clear dose-dependent manner, even the low-dose XFK group significantly narrowed LVIDs and improved EF, FS (*P* < 0.05). Notably, XFK reduced LVIDs but did not significantly change LVIDd, which is consistent with limited reversibility of diastolic chamber size at this post-infarction time point. CO did not display a strictly monotonic dose-dependent manner, likely reflecting its dependence on stroke volume and heart rate, as well as greater variability under isoflurane loading conditions.

Histological analysis supported the echocardiographic findings (Figures 1C,D). HE staining revealed that LADCA ligation caused extensive myofibre rupture and diffuse inflammatory infiltration. These pathological changes were progressively ameliorated by escalating doses of XFK and by captopril. Masson’s trichrome staining demonstrated a pronounced increase in interstitial and replacement fibrosis in the model group (*P* < 0.01), whereas XFK reduced collagen deposition in a clear dose-dependent manner (*P* < 0.01). TUNEL staining showed a significant rise in DNA-fragmented nuclei after LADCA ligation (*P* < 0.01), and XFK lowered the number of TUNEL-positive cells in a dose-dependent manner (*P* < 0.05). Together, these structural findings support the conclusion that XFK mitigates ventricular remodeling, limits fibrosis, and reduces cardiomyocyte loss in CHF.

### 3.2 Evaluation of cell viability and toxicity of XFK in H9c2 cardiomyocytes

**FIGURE 2:**
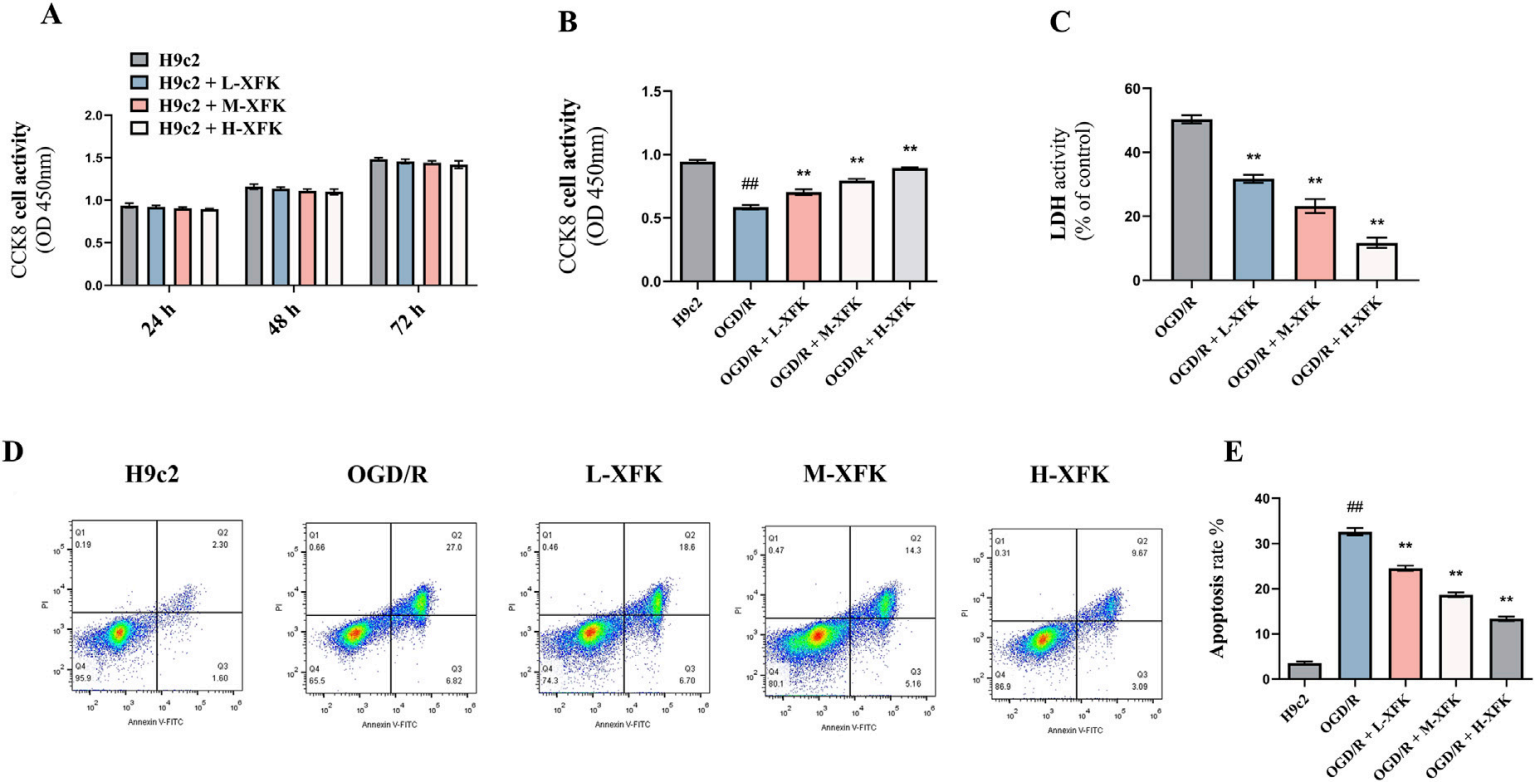
XFK is non-toxic to H9c2 cardiomyocytes and mitigates OGD/R-induced loss of viability, membrane damage, and apoptosis. **(A)** Time-course CCK-8 assay showing that XFK does not affect basal metabolic activity of H9c2 cells at 24 h, 48 h, and 72 h **(B)** CCK-8 assay 24 h after simulated OGD/R demonstrates a pronounced fall in cell viability that is concentration-dependent manner by XFK. **(C)** LDH release rises markedly after OGD/R and is significantly attenuated by escalating doses of XFK. **(D)** Representative Annexin V-FITC/PI flow-cytometric plots illustrating viable (lower-left), early-apoptotic (lower-right), late-apoptotic/necrotic (upper-right), and necrotic (upper-left) populations. **(E)** Quantification of total Annexin V-positive cells (apoptosis rate) confirms a sharp increase after OGD/R that is progressively attenuated by XFK. All data are expressed as mean ± SD, ##*P* < 0.01 vs. H9c2; *P < 0.05, **P < 0.01 vs. OGD/R. L/M/H-XFK (low-, medium-, and high-dose XFK serum).

CCK-8 assays performed at 24 h, 48 h, and 72 h showed that exposure of H9c2 cells to low-, medium-, and high-dose XFK-containing serum did not alter metabolic activity at any time point when compared with untreated controls, indicating an absence of inherent cytotoxicity (Figure 2A). In contrast, simulated OGD/R provoked a marked decline in cell viability (*P* < 0.01). Post-injury administration of XFK increased CCK-8 readings in a concentration-dependent manner, with all three concentrations partially reversing the reduction in CCK-8 absorbance relative to the OGD/R group (*P* < 0.01, Figure 2B). Consistent with these findings, LDH activity was markedly increased after OGD/R, whereas XFK treatment significantly reduced LDH activity in a concentration-dependent manner (*P* < 0.01, Figure 2C). Flow-cytometric Annexin V/propidium iodide staining further demonstrated that OGD/R markedly increased the proportion of Annexin V- and PI-positive cells, whereas escalating concentrations of XFK progressively reduced these populations, supporting its cytoprotective activity (Figure 2D). Collectively, these results indicate that XFK is non-toxic to cardiomyocytes under basal conditions and provides measurable protection against OGD/R-induced loss of viability and membrane integrity. Flow-cytometric quantification of Annexin V/PI staining corroborated the scatter plots, showing a marked rise in apoptotic rate after OGD/R injury that was progressively attenuated by low-, medium-, and high-dose XFK-containing serum (*P* < 0.01, Figure 2E).

### 3.3 XFK attenuates inflammasome-driven inflammation and myocardial pyroptosis in rats with CHF

**FIGURE 3:**
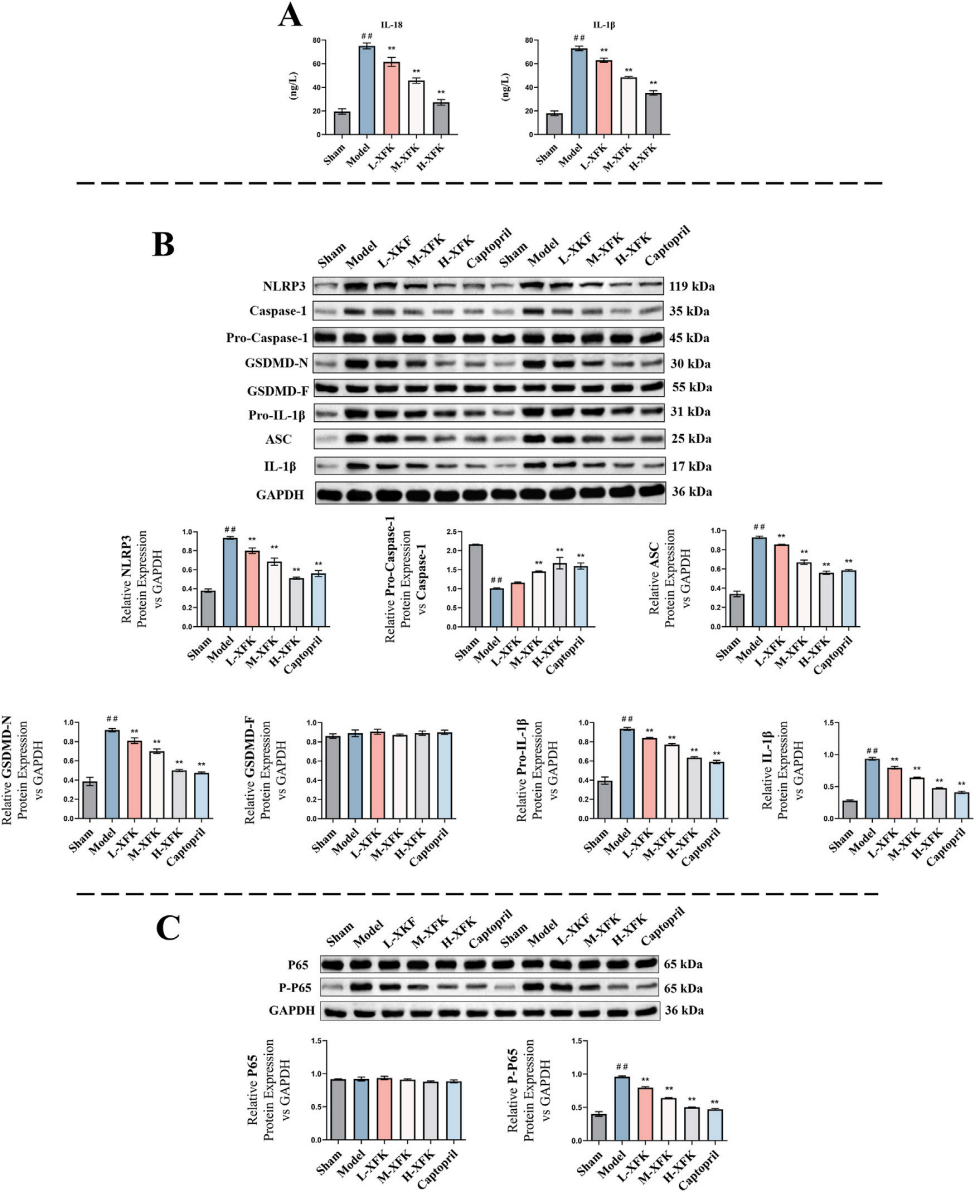
XFK suppresses NLRP3/Caspase-1/GSDMD pyroptotic signaling, pro-inflammatory cytokine release, and NF-κB signaling in CHF rat cardiac tissue. **(A)** ELISA determination of myocardial IL-18 and IL-1β concentrations. LADCA ligation markedly elevated both cytokines, whereas XFK reduced them in a dose-dependent manner. **(B)** Representative immunoblots and corresponding densitometric analyses of NLRP3, pro-caspase-1/caspase-1 ratio, ASC, GSDMD-N, GSDMD-F, pro-IL-1β and IL-1β, with GAPDH as the loading control. CHF markedly upregulated inflammasome components and pyroptosis execution markers; XFK diminished their expression in a dose-dependent manner, with significant effects observed at high doses. **(C)** Immunoblots showing total p65 and phosphorylated p65 and the corresponding quantification. Phosphorylation of p65 was increased in CHF hearts and was selectively suppressed by XFK without altering total p65 abundance. All data are expressed as mean ± SD, ##*P* < 0.01 vs. Sham; *P < 0.05, **P < 0.01 vs. Model. L/M/H-XFK (low-, medium-, and high-dose XFK).

ELISA assays of pyroptosis-associated cytokines showed that CHF markedly increased cardiac tissue concentrations of IL-18 and mature IL-1β when compared with the sham group (*P* < 0.01, Figure 3A). Oral administration of XFK reduced both cytokines in a dose-dependent manner (*P* < 0.01).

Western blot analyses were performed on cardiac tissues collected from rats with CHF induced by LADCA ligation. As shown in Figure 3B, CHF induction significantly elevated the expression of pyroptosis-related proteins, including NLRP3, GSDMD-N, ASC, and IL-1β, and also decreased the pro-caspase-1/caspase-1 ratio compared with the sham group (*P* < 0.01). Treatment with XFK markedly attenuated the protein levels of NLRP3, ASC, GSDMD-N, and IL-1β, and restored the pro-caspase-1/caspase-1 ratio toward sham levels in a dose-dependent manner. These findings indicate that XFK attenuates inflammasome-driven inflammation and myocardial pyroptosis in rats with CHF.

To further investigate upstream signaling, NF-κB pathway activation was assessed. As shown in Figure 3C, phosphorylation of NF-κB p65 was significantly increased in the model group, indicating marked activation of the NF-κB pathway. XFK treatment effectively suppressed p65 phosphorylation without affecting total p65 expression, suggesting inhibition of pathway activation.

### 3.4 XFK attenuates inflammation-related pyroptosis in H9c2 cardiomyocytes following OGD/R injury

To verify the anti-pyroptotic effect of XFK at the cellular level, an *in vitro* OGD/R injury model was established using H9c2 cardiomyocytes.

**FIGURE 4:**
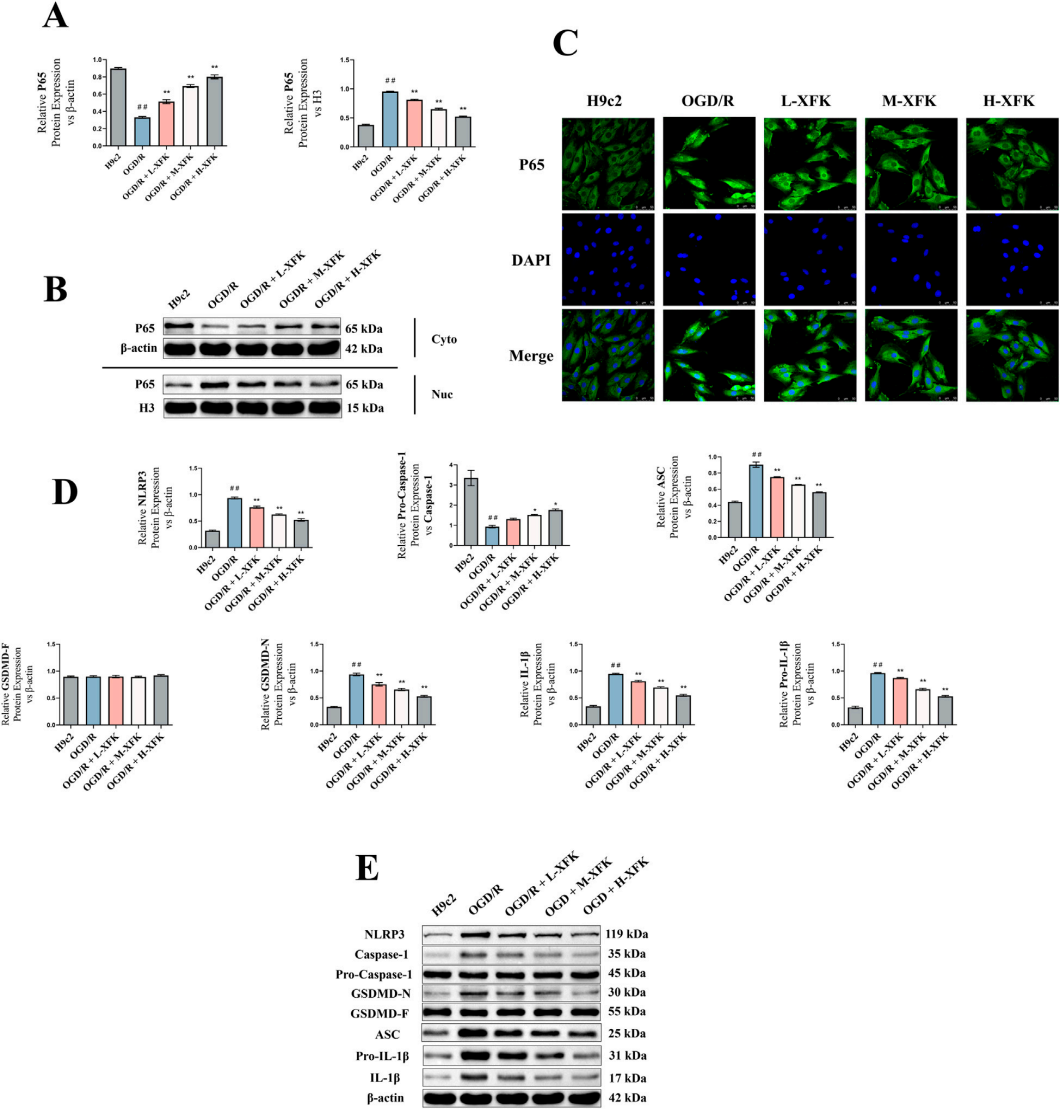
XFK prevents NF-κB nuclear translocation and suppresses NLRP3-inflammasome–dependent pyroptosis in H9c2 cells subjected to *in vitro* OGD/R. **(A)** Densitometric analysis of Western blots showing reciprocal changes in cytoplasmic and nuclear p65 after OGD/R injury. XFK restores cytoplasmic p65 and lowers nuclear p65 in a concentration-dependent manner. **(B)** Representative immunoblots of p65 in cytoplasmic (β-actin loading control) and nuclear (histone H3 loading control) fractions. **(C)** Immunofluorescence images (p65, green; nuclei, DAPI blue) illustrating OGD/R-induced nuclear accumulation of p65 and its progressive reversal by XFK. **(D,E)** Western blot analyses of pyroptosis-related proteins (NLRP3, pro-caspase-1/caspase-1 ratio, ASC, GSDMD-F, GSDMD-N, IL-1β, pro-IL-1β) confirming marked upregulation after OGD/R and concentration-dependent downregulation by XFK. All data are expressed as mean ± SD, ##*P* < 0.01 vs. H9c2; *P < 0.05, **P < 0.01 vs. OGD/R. L/M/H-XFK (low-, medium-, and high-dose XFK serum).

Western blot analysis showed that cytoplasmic p65 levels were decreased and nuclear p65 levels were increased in the OGD/R group compared with the control H9c2 group (*P* < 0.01, Figures 4A,B). XFK treatment resulted in a concentration-dependent increase in cytoplasmic p65 and a concentration-dependent reduction in nuclear p65 levels. Immunofluorescence staining further demonstrated enhanced nuclear translocation of p65 in the OGD/R group, while XFK treatment decreased p65 nuclear translocation in a concentration-dependent manner (Figure 4C). Western blotting further showed that OGD/R markedly increased the expression levels of pyroptosis-related proteins, including NLRP3, ASC, GSDMD-N, and IL-1β, and decreased the pro-caspase-1/caspase-1 ratio (*P* < 0.01), while XFK attenuated these alterations in a concentration-dependent manner (Figures 4D,E).

### 3.5 MiR-223 mediates the protective effect of XFK by inhibiting the NLRP3-related pyroptosis pathway

#### 3.5.1 XFK upregulates miR-223 expression in a concentration-dependent manner

**FIGURE 5:**
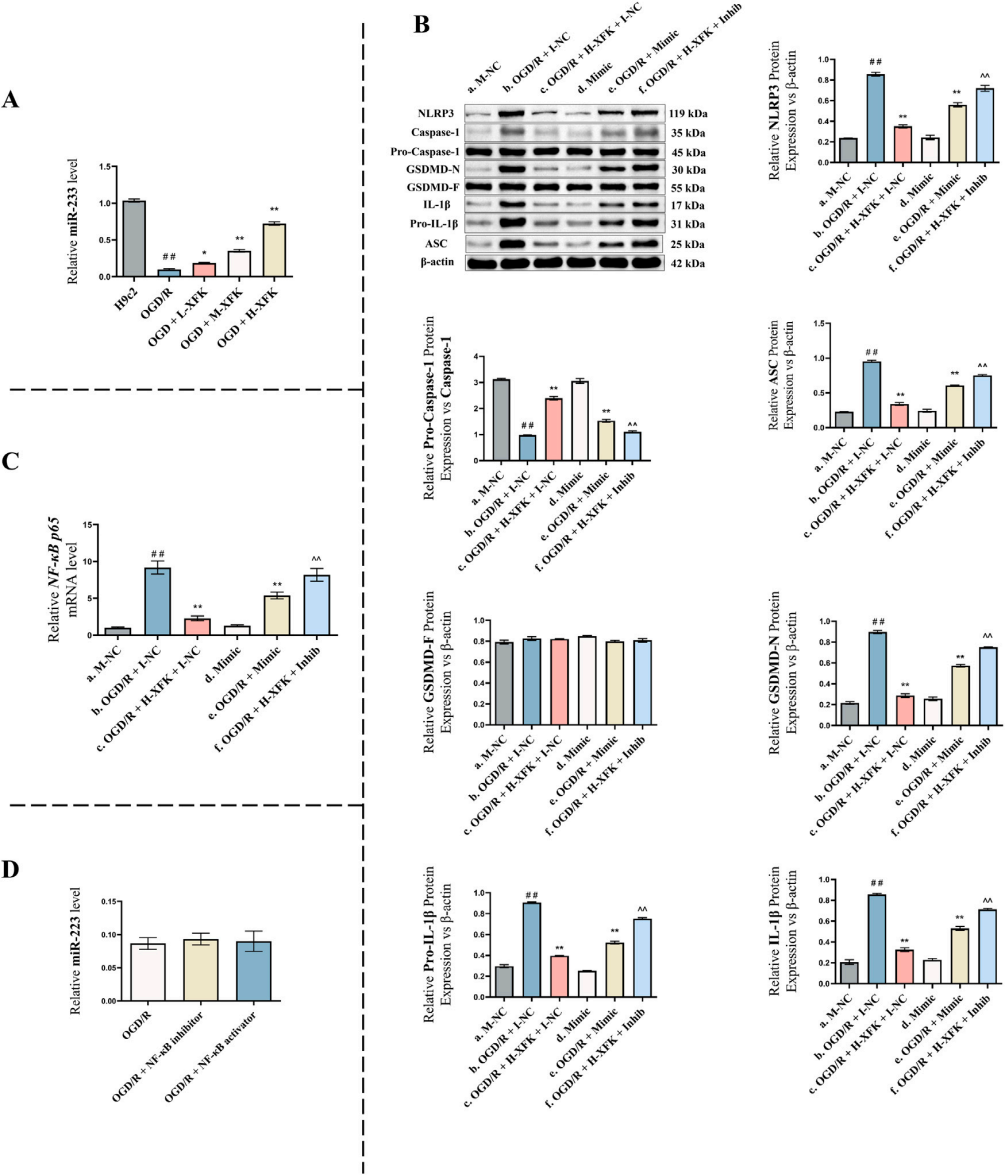
MiR-223 mediates the protective effect of XFK by inhibiting the NLRP3 inflammasome-mediated pyroptosis pathway. **(A)** Relative expression of miR-223 in H9c2 cells subjected to OGD/R injury and treated with different concentrations of XFK. XFK significantly upregulated miR-223 expression in a concentration-dependent manner compared with the OGD/R group. **(B)** Western blot analysis of pyroptosis-related proteins (NLRP3, pro-caspase-1/caspase-1 ratio, ASC, GSDMD-F, GSDMD-N, IL-1β, pro-IL-1β) across different genetic and pharmacologic interventions. **(C)** qPCR analysis of *NF-κB p65* mRNA levels in the same groups as **(B)**. miR-223 overexpression and XFK treatment significantly suppressed *NF-κB p65* mRNA levels induced by OGD/R injury, while miR-223 inhibition reversed the inhibitory effect of XFK. **(D)** qPCR analysis of miR-223 expression following NF-κB pathway inhibition or activation. NF-κB manipulation did not significantly affect miR-223 levels. Data are presented as mean ± SD. In **(A)**, ##*P* < 0.01 vs. H9c2 group; *P < 0.05, **P < 0.01 vs. OGD/R group. In **(B,C)**, #*P* < 0.05, ##*P* < 0.01 vs. Group a; *P < 0.05, **P < 0.01 vs. Group b; ^ *P* < 0.05, ^^ *P* < 0.01 vs. Group c. Mimic/Inhib, miR-223 mimic/inhibitor; M-NC/I-NC, negative controls. L/M/H-XFK (low-, medium-, and high-dose XFK serum).

To explore the role of miR-223 in XFK-mediated protection against pyroptosis, we first examined miR-223 expression under the different treatment conditions used in the OGD/R model. As shown in Figure 5A, treatment with XFK significantly upregulated miR-223 levels in H9c2 cells compared with the OGD/R group, and the effect occurred in a concentration-dependent manner. Low, medium, and high concentrations of XFK produced increasing levels of miR-223 expression across the dosing gradient, with the high-concentration group showing the greatest upregulation (*P* < 0.01). These results indicate that XFK increases miR-223 expression following OGD/R injury.

#### 3.5.2 MiR-223 modulates the expression of pyroptosis-related proteins

To further investigate the functional role of miR-223, rescue experiments were performed (Figure 5B).

Compared with the baseline control (Group a), the expression levels of pyroptosis-related proteins NLRP3, ASC, GSDMD-N, and IL-1β were significantly increased, whereas the pro-caspase-1/caspase-1 ratio was significantly decreased in Group b (a vs. b, *P* < 0.01), indicating the successful establishment of the pyroptotic injury model. Treatment with XFK under the same conditions (Group c) markedly suppressed the expression of these pyroptosis-associated proteins and increased the pro-caspase-1/caspase-1 ratio relative to Group b (c vs. b, *P* < 0.01), suggesting that XFK alleviates OGD/R-induced pyroptosis. Under non-stressed conditions, miR-223 overexpression (Group d) did not significantly alter pyroptosis marker expression compared with Group a (d vs. a, *P* > 0.05), indicating that miR-223 exerts minimal regulatory effects on these markers under basal conditions. However, in OGD/R-injured cells, miR-223 overexpression (Group e) significantly reduced the expression of NLRP3, ASC, GSDMD-N, and IL-1β, and increased the pro-caspase-1/caspase-1 ratio compared with Group b (e vs. b, *P* < 0.01), suggesting that miR-223 exerts a protective effect against pyroptosis under stress conditions. Moreover, miR-223 inhibition in the presence of XFK (Group f) attenuated the protective effects of XFK, leading to significantly higher levels of NLRP3, ASC, GSDMD-N, and IL-1β, together with an decreased pro-caspase-1/caspase-1 ratio, compared with Group c (f vs. c, *P* < 0.01), supporting the conclusion that the anti-pyroptotic action of XFK is at least partly dependent on miR-223.

#### 3.5.3 MiR-223-dependent regulation of *NF-κB p65* mRNA levels under XFK intervention

To determine whether miR-223 mediates the inhibitory effect of XFK on NF-κB signaling, we measured *NF-κB p65* mRNA levels in H9c2 cells across six experimental groups using RT-qPCR (Figure 5C).

Compared with Group a, *NF-κB p65* mRNA levels were significantly upregulated in Group b (a vs. b, *P* < 0.01), consistent with increased NF-κB p65 transcription in response to OGD/R injury. Treatment with XFK (Group c) resulted in a marked reduction in *NF-κB p65* mRNA levels relative to Group b (c vs. b, *P* < 0.01), suggesting that XFK suppresses *NF-κB p65* mRNA expression induced by OGD/R. Under non-stressed conditions, miR-223 overexpression (Group d) did not significantly affect *NF-κB p65* mRNA expression compared with Group a (d vs. a, *P* > 0.05), indicating limited influence on basal NF-κB p65 transcription. However, in OGD/R-injured cells, miR-223 overexpression (Group e) significantly suppressed *NF-κB p65* mRNA expression compared with Group b (e vs. b, *P* < 0.01), suggesting that miR-223 negatively regulates NF-κB p65 transcription under stress conditions. In contrast, miR-223 inhibition under XFK treatment (Group f) attenuated the inhibitory effect of XFK on *NF-κB p65* mRNA expression and resulted in higher *NF-κB p65* mRNA levels compared with Group c (f vs. c, *P* < 0.01), supporting a miR-223-dependent mechanism underlying the anti-inflammatory action of XFK.

#### 3.5.4 NF-κB signaling does not regulate miR-223 expression

To verify the regulatory relationship between NF-κB and miR-223, NF-κB inhibition and activation experiments were conducted (Figure 5D). Compared with the OGD/R group, neither NF-κB inhibition nor NF-κB activation produced any significant change in miR-223 expression (*P* > 0.05). This finding indicate that NF-κB does not function as an upstream regulator of miR-223 under the present experimental conditions, thereby supporting the interpretation that miR-223 influences NF-κB signaling without reciprocal regulation.

## 4 Discussion

**FIGURE 6:**
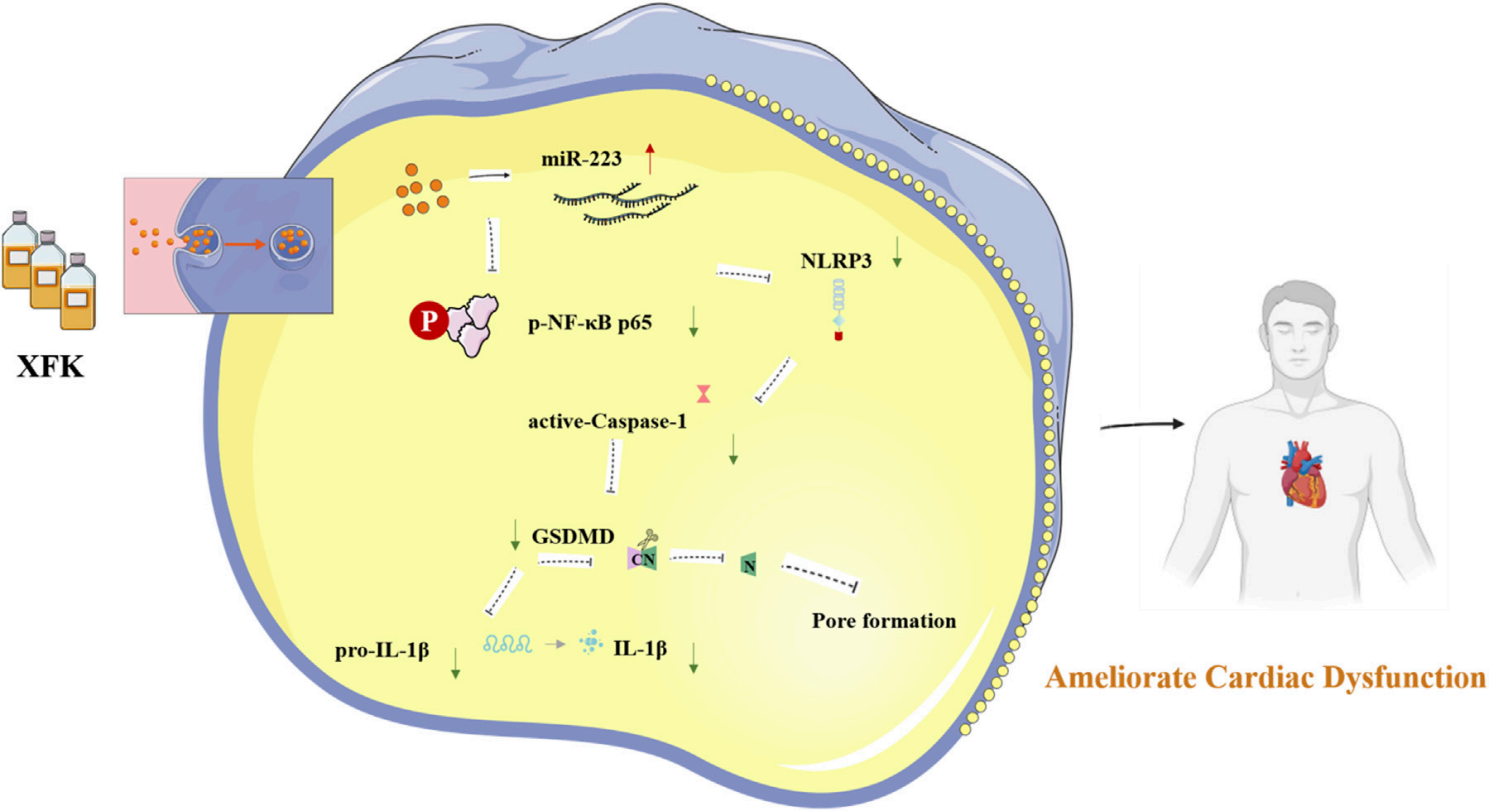
The miR-223-dependent regulatory mechanism by which XFK mitigates NF-κB/NLRP3-associated pyroptosis and improves cardiac function. XFK treatment increases miR-223 expression, which is associated with reduced phosphorylation of NF-κB p65 and downregulation of NLRP3 inflammasome-related signaling. The consequent decrease in caspase-1 activation and GSDMD cleavage limits pore formation and IL-1β maturation, leading to attenuated inflammatory responses and protection against cardiac dysfunction.

XFK administration increased cardiac functional indices and attenuated adverse remodeling in a rat model of CHF. Mechanistically, XFK increased miR-223 expression, leading to suppression of NF-κB activation. This reduced downstream NLRP3 inflammasome signaling, attenuated GSDMD-mediated pyroptosis, and lowered pro-inflammatory cytokine release (Figure 6).

Evidence implicates pyroptosis as a driver of cardiac injury and dysfunction (Toldo and Abbate, 2024; Lan et al., 2025; Duan et al., 2024). This inflammatory death program, mediated by the NLRP3/caspase-1/GSDMD axis, promotes release of IL-1β and IL-18, sustaining inflammation and cardiomyocyte loss associated with adverse ventricular remodeling and progression toward CHF (Chen et al., 2022; Zeng et al., 2020; Zhang L. et al., 2024). In the LADCA-induced CHF model, NLRP3, ASC, GSDMD-N, IL-1β and IL-18 were increased, and the pro-caspase-1/caspase-1 ratio was decreased. XFK reduced each of these molecular indices across tested doses, consistent with suppression of pyroptosis-related signaling.

Evidence indicates that the miR-223–NF-κB axis is involved in upstream regulation of pyroptosis. NF-κB regulates transcription of pro-inflammatory mediators, including NLRP3 and pro-IL-1β, and miR-223 has been reported to constrain NF-κB signaling (Houshmandfar et al., 2021; Zhang et al., 2021). In the present study, XFK increased miR-223 expression. Gain- and loss-of-function experiments showed that miR-223 overexpression reduced NF-κB p65 activation, lowered the levels of NLRP3, ASC, GSDMD-N, and IL-1β, and increased the pro-caspase-1/caspase-1 ratio, whereas miR-223 inhibition attenuated the suppressive effects of XFK on these pyroptosis-related markers. Direct modulation of NF-κB activity did not alter miR-223 expression. Taken together, these findings support a model in which XFK elevates miR-223, leading to reduced NF-κB p65 activity and attenuation of downstream NLRP3 inflammasome signaling.

XFK exerted dose-dependent effects across multiple endpoints, including the suppression of p65 phosphorylation, downregulation of inflammasome proteins, reduction of cytokine output, and inhibition of cell death markers *in vitro*. This bioactivity is likely attributable to the synergistic action of its nine herbal constituents. Many of the major herbs in the XFK formula contain bioactive compounds with anti-inflammatory properties. For example, astragaloside IV from *A. membranaceus* (Huangqi) (Song et al., 2018; Wang et al., 2020; Su et al., 2023), tanshinone IIA from *S. miltiorrhiza* (Danshen) (Chai et al., 2023; Song et al., 2021; Xu et al., 2024), and ginsenosides from *P. ginseng* (Renshen) (Yanna et al., 2022; Jang et al., 2023; Li et al., 2025) have been reported to inhibit NF-κB signaling and reduce inflammatory damage in various cardiovascular disease models. Our findings indicate that the combined activity of these and other constituents within XFK may converges on the miR-223/NF-κB axis to suppress myocardial pyroptosis.

In TCM, the syndrome of “blood stasis” describes a state of impaired circulation, microvascular dysfunction, and pathological retention of blood components that contribute to tissue injury (Wu et al., 2023; Guo et al., 2020; Huang et al., 2023). There is a conceptual parallel between this description and certain aspects of modern understanding of pyroptosis-driven pathology. The exaggerated inflammatory response, endothelial dysfunction, and microcirculatory disturbances associated with pyroptosis-mediated cytokine release and cell death (Cui et al., 2023; Yang et al., 2024; Kel et al., 2025) may represent modern immunopathological correlates of “blood stasis.” From this perspective, XFK, which is prescribed for CHF with “blood stasis,” can be interpreted as a therapy with mechanistic plausibility. By modulating pyroptosis-related inflammatory processes, XFK functions in a manner analogous to a “blood-activating” agent, improving microcirculation and mitigating tissue injury in a way that aligns with its traditional therapeutic principles. XFK holds therapeutic potential as an adjunct to standard CHF regimens. This study suggests XFK may be particularly relevant for the subset of CHF patients characterized by a high-inflammation phenotype, although such implications require clinical investigation before therapeutic applicability can be established.

Previous work from our group and others has shown that XFK confers cardioprotection by improving mitochondrial function and regulating mitophagy through the PI3K/AKT signaling pathway (Zhang et al., 2025; Qiu et al., 2018). The suppression of pyroptosis identified in this study does not contradict these findings but instead provides a complementary mechanistic dimension. Mitochondria are critical sites for inflammasome activation, as mitochondrial dysfunction and the release of mitochondrial reactive oxygen species can promote activation of the NLRP3 pathway. Therefore, by improving mitochondrial function while also modulating the miR-223/NF-κB/NLRP3 axis, XFK may employ an integrated, multi-target strategy for cardioprotection.

## 5 Conclusion

This study suggests that XFK oral liquid may mitigate pathological processes associated with CHF by modulating the miR-223/NF-κB/NLRP3 signaling axis. In association with increased miR-223 expression, XFK resulted in reduced NF-κB activity. It also lowered molecular indices indicative of diminished GSDMD-dependent pyroptosis signaling. These molecular changes were accompanied by improvements in remodeling parameters in the rat CHF model. Taken together, these observations support the potential of XFK as a multi-component adjunct in the context of CHF-related pathology, although further validation in clinical studies is required.

## 6 Research limitations

This study followed a hypothesis-driven, candidate-based design rather than an unbiased transcriptomic screen. We pre-specified miR-223 based on reproducible evidence that it functions as an anti-inflammatory regulator in cardiovascular and innate immune contexts and has been reported to constrain NF-κB signaling and limit NLRP3-related inflammasome activity. Our experiments were therefore structured to test an *a priori* the working hypothesis that XFK mitigates pyroptosis in CHF by upregulating miR-223 and inhibiting NF-κB/NLRP3 related signaling, using gain- and loss-of-function and rescue approaches. We acknowledge that this candidate strategy does not exclude other miRNAs or pathways relevant to pyroptosis. Future work will include unbiased miRNA profiling and direct target validation to complement the present findings.

## Data Availability

The original contributions presented in the study are included in the article/Supplementary Material, further inquiries can be directed to the corresponding authors.

## References

[B1] Aimo A. Castiglione V. Borrelli C. Saccaro L. F. Franzini M. Masi S. (2020). Oxidative stress and inflammation in the evolution of heart failure: from pathophysiology to therapeutic strategies. Eur. J. Prev. Cardiol. 27, 494–510. 10.1177/2047487319870344 31412712

[B2] Chai R. Xue W. Shi S. Zhou Y. Du Y. Li Y. (2022). Cardiac remodeling in heart failure: role of pyroptosis and its therapeutic implications. Front. Cardiovasc Med. 9, 870924. 10.3389/fcvm.2022.870924 35509275 PMC9058112

[B3] Chai R. Ye Z. Xue W. Shi S. Wei Y. Hu Y. (2023). Tanshinone IIA inhibits cardiomyocyte pyroptosis through TLR4/NF-κB p65 pathway after acute myocardial infarction. Front. Cell Dev. Biol. 11, 1252942. 10.3389/fcell.2023.1252942 37766966 PMC10520722

[B4] Chen L. Yin Z. Qin X. Zhu X. Chen X. Ding G. (2022). CD74 ablation rescues type 2 diabetes mellitus-induced cardiac remodeling and contractile dysfunction through pyroptosis-evoked regulation of ferroptosis. Pharmacol. Res. 176, 106086. 10.1016/j.phrs.2022.106086 35033649

[B5] Cui L. Liu Y. Hu Y. Dong J. Deng Q. Jiao B. (2023). Shexiang tongxin dropping pill alleviates M1 macrophage polarization-induced inflammation and endothelial dysfunction to reduce coronary microvascular dysfunction *via* the dectin-1/syk/IRF5 pathway. J. Ethnopharmacol. 316, 116742. 10.1016/j.jep.2023.116742 37290736

[B6] Duan Y. Li Q. Wu J. Zhou C. Liu X. Yue J. (2024). A detrimental role of endothelial S1PR2 in cardiac ischemia-reperfusion injury *via* modulating mitochondrial dysfunction, NLRP3 inflammasome activation, and pyroptosis. Redox Biol. 75, 103244. 10.1016/j.redox.2024.103244 38909407 PMC11254837

[B7] Feng Y. Li L. Zhang Q. Zhang J. Huang Y. Lv Y. (2021). Microtubule associated protein 4 (MAP4) phosphorylation reduces cardiac microvascular density through NLRP3-related pyroptosis. Cell Death Discov. 7, 213. 10.1038/s41420-021-00606-w 34381021 PMC8358013

[B8] Guo R. Li L. Su J. Li S. Duncan S. E. Liu Z. (2020). Pharmacological activity and mechanism of tanshinone IIA in related diseases. Drug Des. Dev. Ther. 14, 4735–4748. 10.2147/DDDT.S266911 33192051 PMC7653026

[B9] Haneklaus M. Gerlic M. O’Neill L. a. J. Masters S. L. (2013). miR-223: infection, inflammation and cancer. J. Intern Med. 274, 215–226. 10.1111/joim.12099 23772809 PMC7166861

[B10] Houshmandfar S. Saeedi-Boroujeni A. Rashno M. Khodadadi A. Mahmoudian-Sani M.-R. (2021). miRNA-223 as a regulator of inflammation and NLRP3 inflammasome, the main fragments in the puzzle of immunopathogenesis of different inflammatory diseases and COVID-19. Schmiedeb. Arch. Pharmacol. 394, 2187–2195. 10.1007/s00210-021-02163-6 34590186 PMC8481106

[B11] Huang Y. Zhang K. Wang X. Guo K. Li X. Chen F. (2023). Multi-omics approach for identification of molecular alterations of QiShenYiQi dripping pills in heart failure with preserved ejection fraction. J. Ethnopharmacol. 315, 116673. 10.1016/j.jep.2023.116673 37268257

[B12] Jang W. Y. Hwang J. Y. Cho J. Y. (2023). Ginsenosides from panax ginseng as key modulators of NF-κB signaling are powerful anti-inflammatory and anticancer agents. Int. J. Mol. Sci. 24, 6119. 10.3390/ijms24076119 37047092 PMC10093821

[B13] Junxiu Z. Yu F. Shaodan L. Yi L. Yin Z. Yunxia G. (2017). Microvascular pathological features and changes in related injury factors in a rat acute blood stasis model. J. Tradit. Chin. Med. 37, 108–115. 10.1016/S0254-6272(17)30034-1 29957919

[B14] Kell D. B. Pretorius E. Zhao H. (2025). A direct relationship between “blood stasis” and fibrinaloid microclots in chronic, inflammatory, and vascular diseases, and some traditional natural products approaches to treatment. Pharm. basel Switz. 18, 712. 10.3390/ph18050712 40430532 PMC12114700

[B15] Lan C. Fang G. Li X. Chen X. Chen Y. Hu T. (2025). SerpinB1 targeting safeguards against pathological cardiac hypertrophy and remodelling by suppressing cardiomyocyte pyroptosis and inflammation initiation. Cardiovasc Res. 121, 113–127. 10.1093/cvr/cvae241 39688818

[B16] Li Y. Zhang M. Zhang K. Niu H. Li H. Wu W. (2025). Ginsenosides modulate immunity via TLR4/MyD88/NF-κB pathway and gut microbiota. Phytomed Int. J. Phytother. Phytopharm. 142, 156763. 10.1016/j.phymed.2025.156763 40252438

[B17] Pan L. Yan B. Zhang J. Zhao P. Jing Y. Yu J. (2022). Mesenchymal stem cells-derived extracellular vesicles-shuttled microRNA-223-3p suppress lipopolysaccharide-induced cardiac inflammation, pyroptosis, and dysfunction. Int. Immunopharmacol. 110, 108910. 10.1016/j.intimp.2022.108910 35978499

[B18] Pfeffer M. A. Pfeffer J. M. Fishbein M. C. Fletcher P. J. Spadaro J. Kloner R. A. (1979). Myocardial infarct size and ventricular function in rats. Circ. Res. 44, 503–512. 10.1161/01.res.44.4.503 428047

[B19] Pfeffer J. M. Pfeffer M. A. Braunwald E. (1985). Influence of chronic captopril therapy on the infarcted left ventricle of the rat. Circulation Res. 57, 84–95. 10.1161/01.RES.57.1.84 3891127

[B20] Piamsiri C. Maneechote C. Jinawong K. Arunsak B. Chunchai T. Nawara W. (2023). GSDMD-mediated pyroptosis dominantly promotes left ventricular remodeling and dysfunction in post-myocardial infarction: a comparison across modes of programmed cell death and mitochondrial involvement. J. Transl. Med. 21, 16. 10.1186/s12967-023-03873-6 36627703 PMC9830763

[B21] Qin J. Yang Q. Wang Y. Shi M. Zhao X. Zhou Y. (2024). The role of pyroptosis in heart failure and related traditional chinese medicine treatments. Front. Pharmacol. 15, 1377359. 10.3389/fphar.2024.1377359 38868667 PMC11168204

[B22] Qiu Z. Hu Y. Geng Y. Wu H. Bo R. Shi J. (2018). Xin fu kang oral liquid inhibits excessive myocardial mitophagy in a rat model of advanced heart failure. Am. J. Transl. Res. 10, 3198–3210. 30416661 PMC6220223

[B23] Roger V. L. (2021). Epidemiology of heart failure: a contemporary perspective. Circ. Res. 128, 1421–1434. 10.1161/CIRCRESAHA.121.318172 33983838

[B24] Shi H. Gao Y. Dong Z. Yang J. Gao R. Li X. (2021). GSDMD-mediated cardiomyocyte pyroptosis promotes myocardial I/R injury. Circ. Res. 129, 383–396. 10.1161/CIRCRESAHA.120.318629 34015941 PMC8291144

[B25] Song M.-T. Ruan J. Zhang R.-Y. Deng J. Ma Z.-Q. Ma S.-P. (2018). Astragaloside IV ameliorates neuroinflammation-induced depressive-like behaviors in mice via the PPARγ/NF-κB/NLRP3 inflammasome axis. Acta Pharmacol. Sin. 39, 1559–1570. 10.1038/aps.2017.208 29795356 PMC6289360

[B26] Song Z. Feng J. Zhang Q. Deng S. Yu D. Zhang Y. (2021). Tanshinone IIA protects against cerebral ischemia reperfusion injury by regulating microglial activation and polarization via NF-κB pathway. Front. Pharmacol. 12, 641848. 10.3389/fphar.2021.641848 33953677 PMC8090935

[B27] Su X. Guo H. Zhou Y. Cao A. Shen Q. Zhu B. (2023). Astragaloside IV attenuates high glucose-induced NF-κB-mediated inflammation through activation of PI3K/AKT-ERK-dependent Nrf2/ARE signaling pathway in glomerular mesangial cells. Phytother. Res. PTR 37, 4133–4148. 10.1002/ptr.7875 37189016

[B28] Sun W. Lu H. Dong S. Li R. Chu Y. Wang N. (2021). Beclin1 controls caspase-4 inflammsome activation and pyroptosis in mouse myocardial reperfusion-induced microvascular injury. Cell Commun. Signal. 19, 107. 10.1186/s12964-021-00786-z 34732218 PMC8565084

[B29] Toldo S. Abbate A. (2024). The role of the NLRP3 inflammasome and pyroptosis in cardiovascular diseases. Nat. Rev. Cardiol. 21, 219–237. 10.1038/s41569-023-00946-3 37923829 PMC11550901

[B30] Wang N. Zhang X. Ma Z. Niu J. Ma S. Wenjie W. (2020). Combination of tanshinone IIA and astragaloside IV attenuate atherosclerotic plaque vulnerability in ApoE(-/-) mice by activating PI3K/AKT signaling and suppressing TRL4/NF-κB signaling. Biomed. Pharmacother. = Biomed. Pharmacother. 123, 109729. 10.1016/j.biopha.2019.109729 31887543

[B31] Wu S. Sun Z. Guo Z. Li P. Mao Q. Tang Y. (2023). The effectiveness of blood-activating and stasis-transforming traditional chinese medicines (BAST) in lung cancer progression-a comprehensive review. J. Ethnopharmacol. 314, 116565. 10.1016/j.jep.2023.116565 37172918

[B32] Xin Q. Chen X. Yuan R. Yuan Y. Hui J. Miao Y. (2021). Correlation of platelet and coagulation function with blood stasis syndrome in coronary heart disease: a systematic review and meta-analysis. Chin. J. Integr. Med. 27, 858–866. 10.1007/s11655-021-2871-2 34532747

[B33] Xiong Y. Zhang Z. Liu S. Shen L. Zheng L. Ding L. (2024). Lupeol alleviates autoimmune myocarditis by suppressing macrophage pyroptosis and polarization via PPARα/LACC1/NF-κB signaling pathway. Phytomedicine 123, 155193. 10.1016/j.phymed.2023.155193 37976692

[B34] Xu Z. Cai K. Su S.-L. Zhu Y. Liu F. Duan J.-A. (2024). Salvianolic acid B and tanshinone IIA synergistically improve early diabetic nephropathy through regulating PI3K/akt/NF-κB signaling pathway. J. Ethnopharmacol. 319, 117356. 10.1016/j.jep.2023.117356 37890803

[B35] Xu J. Kang L. Tai B. Liu C. Zhang Z. Ding Q. (2025). The stems of Syringa oblata Lindl. exert cardioprotective effects against acute myocardial ischemia by inhibiting the TLR4/MyD88/NF-κB and NLRP3 inflammasome signaling pathways in mice. J. Ethnopharmacol. 344, 119563. 10.1016/j.jep.2025.119563 40015537

[B36] Yanna L. Linlin Q. Shichao W. Yingchun L. Daidi F. (2022). Ginsenoside Rk1 prevents UVB irradiation-mediated oxidative stress, inflammatory response, and collagen degradation via the PI3K/AKT/NF-κB pathway *in vitro* and *in vivo* . Pubmed 70, 15804–15817. 10.1021/acs.jafc.2c06377 36472249

[B37] Yan Y. Lu K. Ye T. Zhang Z. (2019). MicroRNA-223 attenuates LPS-Induced inflammation in an acute lung injury model *via* the NLRP3 inflammasome and TLR4/NF-κB signaling pathway via RHOB. Int. J. Mol. Med. 43, 1467–1477. 10.3892/ijmm.2019.4075 30747229 PMC6365085

[B38] Yang Y. Zhu Y. Liu C. Cheng J. He F. (2024). Taohong siwu decoction reduces acute myocardial ischemia-reperfusion injury by promoting autophagy to inhibit pyroptosis. J. Ethnopharmacol. 321, 117515. 10.1016/j.jep.2023.117515 38042386

[B39] Yu G. Wang J. (2014). Blood stasis syndrome of coronary heart disease: a perspective of modern medicine. Chin. J. Integr. Med. 20, 300–306. 10.1007/s11655-013-1332-3 23893237

[B40] Zeng C. Duan F. Hu J. Luo B. Huang B. Lou X. (2020). NLRP3 inflammasome-mediated pyroptosis contributes to the pathogenesis of non-ischemic dilated cardiomyopathy. Redox Biol. 34, 101523. 10.1016/j.redox.2020.101523 32273259 PMC7327979

[B41] Zhang L.-Z. Xue H. Qiao C.-X. You W.-L. Di A.-T. Zhao G. (2021). MiR-223 promotes pyroptosis of enteritis cells through activating NF-κB signalling pathway by targeting SNIP1 in inflammatory bowel disease. Taylor Fr Autoimmunity 54, 362–372. 10.1080/08916934.2021.1940973 34151668

[B42] Zhang Z. Yang Z. Wang S. Wang X. Mao J. (2024). Overview of pyroptosis mechanism and in-depth analysis of cardiomyocyte pyroptosis mediated by NF-κB pathway in heart failure. Biomed. Pharmacother. = Biomed. Pharmacother. 179, 117367. 10.1016/j.biopha.2024.117367 39214011

[B43] Zhang L. Li Y. Fan C.-D. Jiang Y.-H. Sheng L.-S. Song X.-Y. (2024). Chinese medicinal formula fu xin decoction against chronic heart failure by inhibiting the NLRP3/caspase-1/GSDMD pyroptotic pathway. Biomed. Pharmacother. = Biomed. Pharmacother. 174, 116548. 10.1016/j.biopha.2024.116548 38599064

[B44] Zhang X. Chang X. Chai R. Zhang X. Li J. Guo Z. (2025). Xin-fu-kang oral liquid mitigates chronic heart failure through NR4A1-dependent regulation of endoplasmic reticulum-mitochondrial crosstalk in cardiomyocytes. Phytomedicine 140, 156467. 10.1016/j.phymed.2025.156467 40036990

