# Supplementary material for: Xin-Fu-Kang oral liquid improves cardiac function and attenuates miR-223–associated NF-κB/NLRP3 pyroptotic signaling in chronic heart failure
Source: Front Pharmacol. 2025 Dec 11;16:1697422. doi: 10.3389/fphar.2025.1697422 (PMC12738884; doi:10.3389/fphar.2025.1697422)
Supplement: Supplementary file 1 [file Table1.docx]

**Supplementary Table S1. qPCR oligonucleotides used in this study (5′→3′).**

| **Target** | **Primer name** | **Sequence (5′→3′)** | **Primer type / orientation** |
| --- | --- | --- | --- |
| rno-miR-223-3p | miR-223-3p Forward | GTGCTCCGTGTCAGTTTGTCA | miRNA-specific forward primer |
|  | Universal Reverse (kit) | TCGTATCCAGTGCAGGGTC | Universal reverse primer |
|  | Stem-loop RT Primer | GTCGTATCCAGTGCAGGGTCCGAGGTATTCGCACTGGATACGACGGGGTATT | Stem-loop reverse-transcription primer |
| NF-κB p65 | RELA Forward | CGTGAGGCTGTTTGGTTTGA | mRNA forward primer |
|  | RELA Reverse | CTGTCTTATGGCTGAGGTCTGG | mRNA reverse primer |

Notes: miRNA assays used a stem-loop RT design; the “Universal Reverse (kit)” primer was provided with the miRNA cDNA/quantification kit.
