# Supplementary figures and images for: Xin-Fu-Kang oral liquid improves cardiac function and attenuates miR-223–associated NF-κB/NLRP3 pyroptotic signaling in chronic heart failure
Source: Front Pharmacol. 2025 Dec 11;16:1697422. doi: 10.3389/fphar.2025.1697422 (PMC12738884; doi:10.3389/fphar.2025.1697422)

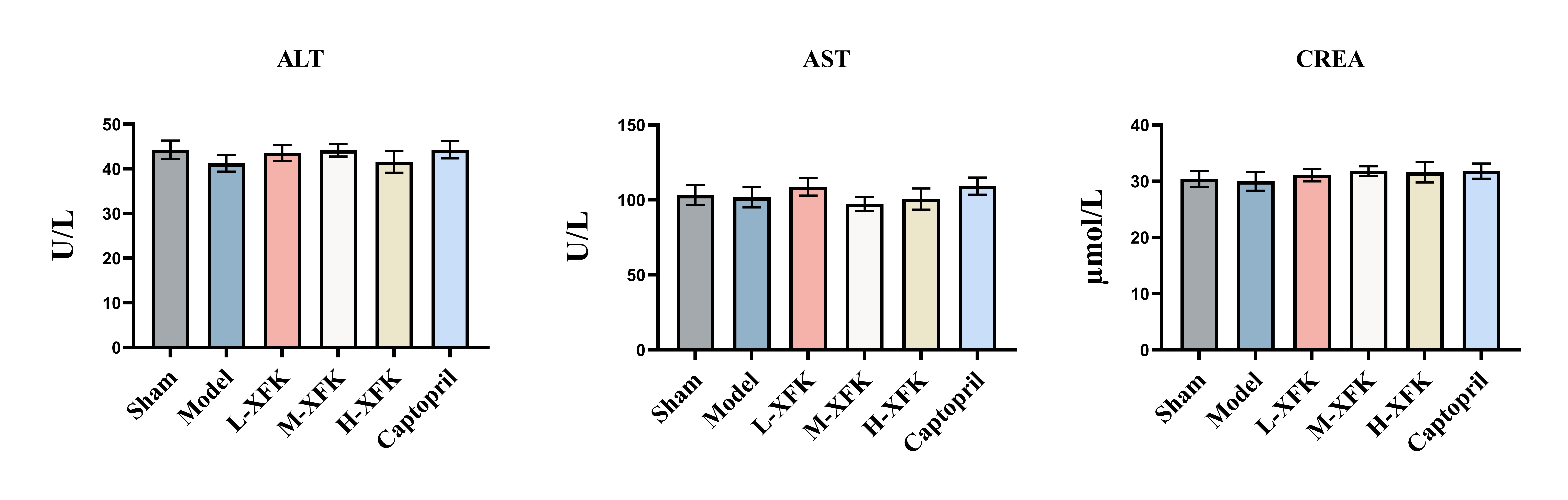

Supplement: Supplementary file 2 [file Image1.tif]
