# Supplementary material for: Xin-Fu-Kang oral liquid improves cardiac function and attenuates miR-223–associated NF-κB/NLRP3 pyroptotic signaling in chronic heart failure
Source: Front Pharmacol. 2025 Dec 11;16:1697422. doi: 10.3389/fphar.2025.1697422 (PMC12738884; doi:10.3389/fphar.2025.1697422)
Supplement: Supplementary file 3 [file DataSheet1.docx]

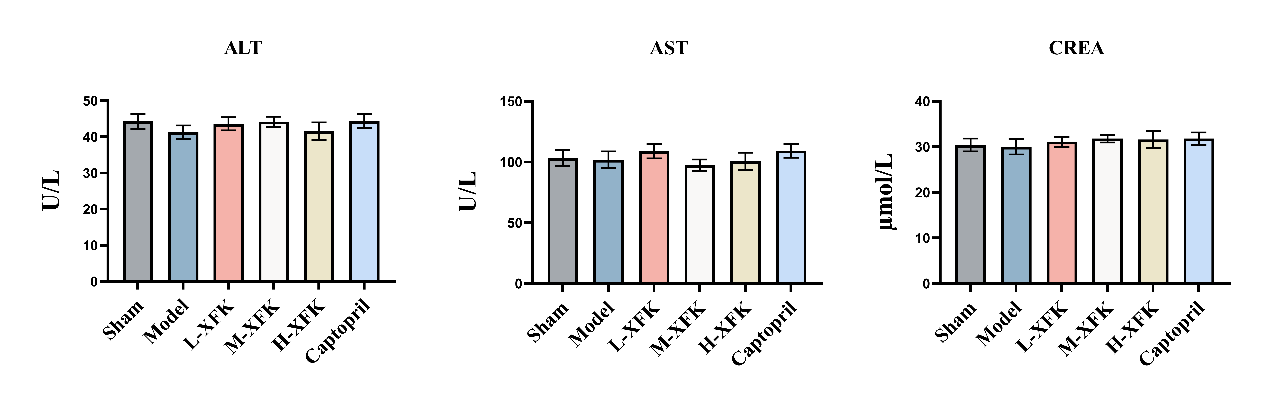


**Figure S1. XFK does not induce hepatic or renal toxicity in rats.**
Serum alanine aminotransferase (ALT) activities in Sham, Model, and XFK-treated groups (L-XFK, M-XFK, H-XFK), as well as the Captopril group. ALT levels remained within the physiological range across all groups, with no significant differences, indicating preserved hepatocellular integrity.
Serum aspartate aminotransferase (AST) activities showing comparable values among all treatment groups. OGD/R injury or XFK administration did not alter AST activity, further supporting the absence of hepatocellular damage.
Serum creatinine (CREA) concentrations demonstrating stable renal function in all groups. Neither the disease model nor XFK treatment at any dose affected creatinine levels.
All data are expressed as mean ± SD. Sham, Model, L/M/H-XFK (low-, medium-, and high-dose XFK).
